# 4-hydroxytamoxifen does not deteriorate cardiac function in cardiomyocyte-specific MerCreMer transgenic mice

**DOI:** 10.1007/s00395-020-00841-9

**Published:** 2021-02-05

**Authors:** Andre Heinen, Stefanie Gödecke, Ulrich Flögel, Dominika Miklos, Katharina Bottermann, André Spychala, Axel Gödecke

**Affiliations:** 1 Institut für Herz- und Kreislaufphysiologie, Medizinische Fakultät und Universitätsklinikum Düsseldorf, Heinrich-Heine-Universität Düsseldorf, Universitätsstraße 1, 40225 Düsseldorf, Germany; 2 Institut für Molekulare Kardiologie, Medizinische Fakultät und Universitätsklinikum Düsseldorf, Heinrich-Heine-Universität Düsseldorf, 40225 Düsseldorf, Germany

**Keywords:** aMHC-MerCreMer/loxP system, 4-hydroxytamoxifen, Cardiac function, Cardiomyopathy, Cardiac energetics

## Abstract

Conditional, cell-type-specific transgenic mouse lines are of high value in cardiovascular research. A standard tool for cardiomyocyte-restricted DNA editing is the αMHC-MerCreMer/loxP system. However, there is an ongoing debate on the occurrence of cardiac side effects caused by unspecific Cre activity or related to tamoxifen/oil overload. Here, we investigated potential adverse effects of DNA editing by the αMHC-MerCreMer/loxP system in combination with a low-dose treatment protocol with the tamoxifen metabolite 4-hydroxytamoxifen (OH-Txf). αMHC-MerCreMer mice received intraperitoneally OH-Txf (20 mg/kg) for 5 or 10 days. These treatment protocols were highly efficient to induce DNA editing in adult mouse hearts. Multi-parametric magnetic resonance imaging revealed neither transient nor permanent effects on cardiac function during or up to 19 days after 5 day OH-Txf treatment. Furthermore, OH-Txf did not affect cardiac phosphocreatine/ATP ratios assessed by in vivo ^31^P MR spectroscopy, indicating no Cre-mediated side effects on cardiac energy status. No MRI-based indication for the development of cardiac fibrosis was found as mean T1 relaxation time was unchanged. Histological analysis of myocardial collagen III content after OH-Txf confirmed this result. Last, mean T2 relaxation time was not altered after Txf treatment suggesting no pronounced cardiac lipid accumulation or tissue oedema. In additional experiments, cardiac function was assessed for up to 42 days to investigate potential delayed side effects of OH-Txf treatment. Neither 5- nor 10-day treatment resulted in a depression of cardiac function. Efficient cardiomyocyte-restricted DNA editing that is free of unwanted side effects on cardiac function, energetics or fibrosis can be achieved in adult mice when the αMHC-MerCreMer/loxP system is activated by the tamoxifen metabolite OH-Txf.

**Supplementary Information:**

The online version of this article (10.1007/s00395-020-00841-9) contains supplementary material, which is available to authorized users.

## Introduction

Conditional gene modification technologies are widely used in the field of cardiovascular research to study physiological and pathophysiological gene functions in mouse models. A common tool for conditional gene modification including induction of gene knockout or overexpression as well as cell lineage fate mapping is the Cre/loxP system. Here, the Cre (causes recombination) recombinase induces site-specific DNA recombination between two loxP (locus of crossover (x) P1 bacteriophage) sites, and thereby causes deletion of the DNA sequences flanked by the loxP sites. However, constitutive gene deletion, often causes embryonal lethality. To overcome this limitation, temporally controlled gene editing is employed by which the gene deletion is induced in the adult mouse. A commonly used tool to achieve a temporal control is the fusion of the Cre-recombinase with mutated-hormone-binding sites of the oestrogen or progesterone receptors allowing the control of Cre activity by the injection of the receptor ligands, such as tamoxifen, raloxifen and RU486, respectively [25, 34]. Among the binding sites, those derived from the estrogenic receptor are the most frequently used for temporal control of Cre activity (Cre-ERT, Cre-ERT2, MerCreMer). The point mutations alter the ligand-binding properties in that the mutated sites have a higher affinity to synthetic ligands, such as tamoxifen, and a reduced affinity for the natural ligand oestrogen. In addition to this temporal control, the use of tissue-specific promotors to drive inducible Cre expression in mice with loxP-containing genes allows spatial control of mutagenesis. For example, the α-myosin heavy chain promotor (αMHC-MerCreMer) allows cardiomyocyte-restricted DNA editing [31]. Although this αMHC-MerCreMer/loxP system is used in many research laboratories, there is an ongoing debate on potential cardiac site effects that would limit the applicability of this gene-editing strategy. Whereas the original work from the Molkentin group investigating the αMHC-MerCreMer/loxP system does not show detrimental effects on cardiac function [31], other studies report on the occurrence of systolic dysfunction, disturbed cardiac energetics, and cardiomyopathy, which were dependent on Cre-activation [12, 20]. In rat cardiomyocytes, Cre-independent effects of tamoxifen as well as its active metabolite 4-hydroxytamoxifen (OH-Txf) on contraction and Ca^2+^-handling have been described [2]. Interestingly, both compounds given at equimolar concentrations affected cardiomyocyte function to a comparable extent. However, because OH-Txf is the active metabolite of tamoxifen which has a much higher affinity to the oestrogen receptor compared to tamoxifen, the question raises whether cardiac side effects can be reduced or avoided using lower doses of OH-Txf compared to tamoxifen.

Therefore, the aim of the present study was to investigate the potential adverse effects of OH-Txf-induced activation of the Cre-recombinase in αMHC-MerCreMer mice on cardiac function using different treatment protocols of OH-Txf application that sufficiently induce cardiac DNA editing. For this, cardiac function, energy status and tissue characteristics were analysed in αMHC-MerCreMer mice during OH-Txf treatment using a combined in vivo approach of echocardiography and multi-parametric resonance imaging/spectroscopy.

## Methods

### Animal experiments

All animal experiments were performed conforming to the guidelines from Directive 2010/63/EU of the European Parliament on the protection of animals used for scientific purposes after approval of the Bezirksregierung Düsseldorf, Germany. Mice were housed in conventional cages with a 12-h light/dark cycle and had ad libitum access to food and water. At the end of the observational period, mice were sacrificed by cervical dislocation. All experiments were performed in a randomized manner, and the analyses of all experimental data were conducted by investigators that where blinded to the treatment protocol.

### Treatment protocols

1. To demonstrate the efficiency of the αMHC-MerCreMer/loxP system in combination with OH-Txf, αMHC-MerCreMer mice carrying floxed alleles of either protein kinase B isoforms 1 and 2 (iCM-Akt1/2), glycogen synthase kinase 3β (iCM-GSK3β), or p38 mitogen-activated kinases (iCM-p38) were used. OH-Txf (Sigma H6278, Munich, Germany) was dissolved at a concentration of 5 mg/ml. To prepare OH-Txf solution, 50 mg OH-Txf was thawed to room temperature for 6–8 h. Subsequently, 200 µl EtOH (100%, 37 °C) was added and the suspension was vortexed. 5 ml pre-warmed peanut oil (Sigma P2144, Munich, Germany, 37 °C) was added, and the suspension was sonicated for 10 min. Finally, 4.8 ml pre-warmed peanut oil was added and sonicated (10–20 min) until OH-Txf was completely dissolved. The solution was stored in aliquots at − 20 °C. For knockout induction, iCM-Akt1/2, iCM-Gsk3β or iCM-p38 mice aged 3–4 month received OH-Txf intraperitoneally (20 mg/kg) for either 5, 7, or 10 consecutive days, respectively, and hearts were excised two weeks after the end of the OH-Txf treatment for western blot analysis of protein depletion. In addition, immunohistological stainings were performed of hearts from iCM-Akt1/2KO or WT mice two weeks after 5 day OH-Txf treatment.
2. The investigations on potential cardiac side effects were performed using male αMHC-MerCreMer mice [31, 34], (C57Bl/6 J background; no loxP sites) aged 3–5 month. Mice received OH-Txf intraperitoneally (20 mg/kg) for five days, and consecutive in vivo multi-parametric cardiac magnetic resonance (MR) imaging and spectroscopy was performed to assess cardiac function and energetic status. Additionally, as it has been demonstrated that myocardial fibrosis, and tissue oedema/lipid accumulation are accompanied by alterations in T1 and T2 relaxation time, respectively [13, 17, 27], these parameters were used as markers for the potential development of cardiac fibrosis, and tissue oedema/lipid accumulation.
3. To further investigate whether OH-Txf treatment causes functional defects at later time points, cardiac function of male αMHC-MerCreMer mice aged 3–4 month was assessed by echocardiography before the start of a five-day OH-Txf treatment and after 3 and 6 weeks. Furthermore, to investigate potential functional side effects of a prolonged 10 day treatment with OH-Txf, cardiac function was assessed before and after 1, 2, 3, and 6 weeks after the start of the OH-Txf treatment.

### Cardiac magnetic resonance imaging and spectroscopy

*General* All data were recorded on a Bruker Avance^III^ 9.4 T Wide Bore (89 mm) nuclear MR spectrometer operating at frequencies of 400.2 MHz for ^1^H and 162.0 MHz for ^31^P measurements essentially as described [7, 33]. Experiments were carried out using a Bruker microimaging unit (Micro 2.5) equipped with actively shielded gradient sets (capable of 1.5 T/m maximum gradient strength and 150 μs rise time at 100% gradient switching), a dual tuneable ^1^H/^31^P 25-mm birdcage resonator, and Paravision 5.1 as operating software. The mice were anesthetized with 1.5% isoflurane and were kept at 37 °C. The front paws and the left hind paw were attached to ECG electrodes (Klear-Trace; CAS Medical Systems, Branford) and respiration was monitored by means of a pneumatic pillow positioned at the animal’s back. Vital functions were acquired by a M1025 system (SA Instruments, Stony Brook, NY, USA) and used to synchronize data acquisition with cardiac and respiratory motion.

*Functional and morphometric analysis* was carried out essentially as described [11] using an ECG- and respiratory-gated segmented fast gradient echo (GE) cine sequence with steady-state precession (FISP). A flip angle of 15°, echo time (TE) of 1.2 ms, and a repetition time (TR) of about 6–8 ms (depending on the heart rate) were used to acquire 16 frames per heart cycle. The pixel size after zero filling was 117 × 117 μm^2^ (field of view, 30 × 30 mm^2^; acquisition time per slice for one cine sequence, ~ 1 min). Routinely, 8–10 contiguous short-axis slices were required for complete coverage of the LV, which was ensured by longitudinal slices orientated perpendicular to the atrio-ventricular level. For evaluation of functional parameters (e.g. EDV, ESV, EF), ventricular demarcations in end-diastole and -systole are manually drawn with the ParaVision Region-of-Interest (ROI) tool.

*Cardiac tissue characterization* was performed by T1 and T2 mapping as described previously [5, 11]. For T1 mapping, we used retrospective triggering with variable flip angle analysis (2°, 5°, 8°, 11°, and 14°), while T2 maps were acquired using an ECG and respiratory-gated multi-echo sequence with 16 echos, separated by a ΔTE of 3.36 ms. *CrCEST* was carried out as essentially reported [14] and slightly modified as described previously for visualization of glycosoaminoglycans [8, 29]. To adapt the timing for an efficient saturation transfer to the rapid heart cycle of the mouse (100–120 ms) and to avoid potential contaminations from ‘CEST-active’ components in the blood (in particular sugars and glycosylated compounds), an ECG- and respiratory-gated GE sequence with an initial ‘black blood preparation’ was implemented as reported in detail recently [29]. Magnetization transfer ratio asymmetry (MTR_asym_) maps were calculated on a pixelwise basis from a series of GE images acquired after black blood preparation and presaturation (6.0 µT, 109.6 Hz,) at ± 700 Hz (S_(±ω)_; ± 1.75 ppm at 9.4 T) and + 5000 Hz (S_(0)_) from the water signal, respectively, using an in-house developed software module as described before6 with MTR_asym(ω)_ = (*S*_(-ω)_ −* S*_(+ω)_)/S_(0)_ [%]. Only intensities from the left ventricular myocardium were used for calculation of the mean CEST contrast.

Subsequently, *cardiac high energy phosphates* were monitored using 2D ^31^P chemical shift imaging (CSI). The slice used for spectroscopic imaging (6 mm) was placed mid-ventricular in short-axis orientation covering almost the entire heart from apex to the base. Fieldmap-based shimming (MAPSHIM) was carried out to optimize the field homogeneity in the region of interest. The 2D ^31^P CSI data set was recorded with a sine-bell acquisition-weighted sequence to improve the spatial response function using the following parameters: flip angle, 45°; TR, 250 ms; field of view, 30 × 30 mm^2^; matrix 16 × 16 (voxel size ~ 20 μl); data points in the spectral domain, 1024; spectral width, 6510 Hz; slice selection with a 500-μs sinc3 pulse; acquisition time, 66 min. Data sets were analysed by an in-house-developed software module based on the LabVIEW package (National Instruments, Austin) [7]. An exponential filter of 20 Hz was applied in the spectroscopic direction and chemical shifts were referenced to the PCr resonance at -2.52 ppm. For quantification of myocardial PCr/ATP ratios, only voxels covering the free left ventricular wall were considered, since in both groups, spectra of septal voxels were occasionally contaminated with ^31^P signals originating from chamber blood as reflected by the appearance of 2,3-diphosphoglycerate (DPG) signals.

### Echocardiography

Left ventricular function was analysed by echocardiography before the start OH-Txf treatment (d0), and subsequently either after 21 and 42 days (d21 and d42, respectively) in mice treated with OH-Txf for five days, or after 7, 14, 21 and 42 days (d7, d14, d21 and d42, respectively) in mice treated with OH-Txf for ten days. Echocardiography was performed using a Vevo2100 system (Visualsonics) equipped with a 30 MHz linear scanner as described previously [16]. In brief, measurements were conducted at 37 °C under isoflurane (2.5%) anaesthesia. Brightness (B)-mode movies of the parasternal long axis and three orthogonal short-axis views (mid-ventricular, apical, and basal) were acquired by a blinded investigator, and post-acquisition analysis was performed by the same investigator. For analysis of end diastolic and end systolic volumes, the endocardium of the left ventricle was traced at both diastole and systole using an integrated software tool (Simpson). Ejection fraction was calculated using the formula EF = ((EDV-ESV)/EDV)*100. Parasternal short-axis M-mode views of the mid-ventricular level were used for determination of wall thickness, dimensions, and weight of the left ventricle.

### Protein analysis

Protein analyses from heart samples were performed as described previously [15]. In brief, whole hearts were homogenized, proteins were separated by SDS-PAGE on polyacrylamide gels, and transferred onto Protran nitrocellulose membranes (GE Healthcare). Membranes were incubated with primary antibodies against AKT1 (#2967), AKT2 (#3063), GSK3β (#9315), and GAPDH (#2118) from Cell Signaling Technology, or p38 (ab170099) from Abcam. Secondary antibodies used were α-rabbit or α-mouse IRDye800CW and α-rabbit or α-mouse IRDye680RD from LI-COR Biosciences. Signals were detected and quantified with an Odyssey near-infrared scanner (LI-COR Biosciences).

### RNA isolation, cDNA synthesis and real-time qPCR

Total RNA was isolated from heart tissue of the area at risk using the Fibrous Tissue RNeasy Kit (QIAGEN, Hilden, Germany; 74,704) according to the manufacturer’s instructions. cDNA was synthesized from the RNA using the QuantiTect reverse transcription kit (QIAGEN, 205,313). qPCR was performed on the Step-One Plus real-time PCR system (Applied Biosystems) with Maxima SYBR Green and ROX qPCR Master Mix (Thermo Scientific). Transcript quantities were analyzed by the X0 method, and normalized to *Actb* mRNA. The following PCR primer sequences were used: *Actb* forward GATGTATGAAGGCTTTGGTC, reverse: TGTGCACTTTTATTGGTCTC; *Nppa* forward GAGAGAAAGAAACCAGAGTG, reverse GTCTAGCAGGTTCTTGAAATC; *Nppb* forward AATTCAAGATGCAGAAGCTG, reverse GAATTTTTGAGGTCTCTGCTG; *Atp2a2* forward AATTGGAGAAGTGCAAAAGG, reverse TACATTCATCTTCTCCACCAG.

### Immunohistology

Immunofluorescence staining of cardiac cryosections was performed as described previously with slight modifications [6, 15]. For the determination of cardiac fibrosis, Masson trichrome staining was performed on cardiac tissue slices (4 μm, short-axis orientation, mid-ventricular level) as described previously [15]. Alternatively, cardiac slices were incubated with an anti-collagen III antibody (ab7778) from Abcam overnight at 4 °C. Secondary antibody (Cy3 AffiniPure goat anti-rabbit immunoglobulin G (IgG);111–165-144) was incubated for 3 h at room temperature in the dark.

For immunofluorescence staining of AKT in the myocardium, iCM-Akt1/2KO or WT mice were starved for 4 h, and received intraperitoneally insulin (3 IU/kg) 15 min before organ harvesting. Wheat germ agglutinin (Lectin from *triticum vulgaris* FITC conjugate; # L4895, Sigma-Aldrich, St. Louis, MO, USA)-labelled 8 μm slices were stained with either pAkt1 (Ser473; Cell Signalling #9018) or pAkt2 (S474; Cell Signalling #8599) antibody. Secondary antibody (Cy3 AffiniPure goat anti-rabbit immunoglobulin G (IgG);111–165-144) was incubated for 3 h at room temperature in the dark. The slides were mounted with DAPI Fluoromount-G (SouthernBiotech). Analyses were carried out using a Keyence immunofluorescence microscope (BZ 9000) and ImageJ software [30].

### Statistical analysis

All data are presented as mean ± SD. The statistical analysis was performed using SigmaPlot 13.0 (Systat software, San Jose, USA). Data were analysed by unpaired, two-tailed student’s T-test or one-way repeated-measure ANOVA, followed by Tukey’s post hoc test. A p-value < 0.05 was considered significant.

## Results

### OH-Txf causes efficient gene deletion in floxed αMHC-MerCreMer mice

**Fig. 1:**
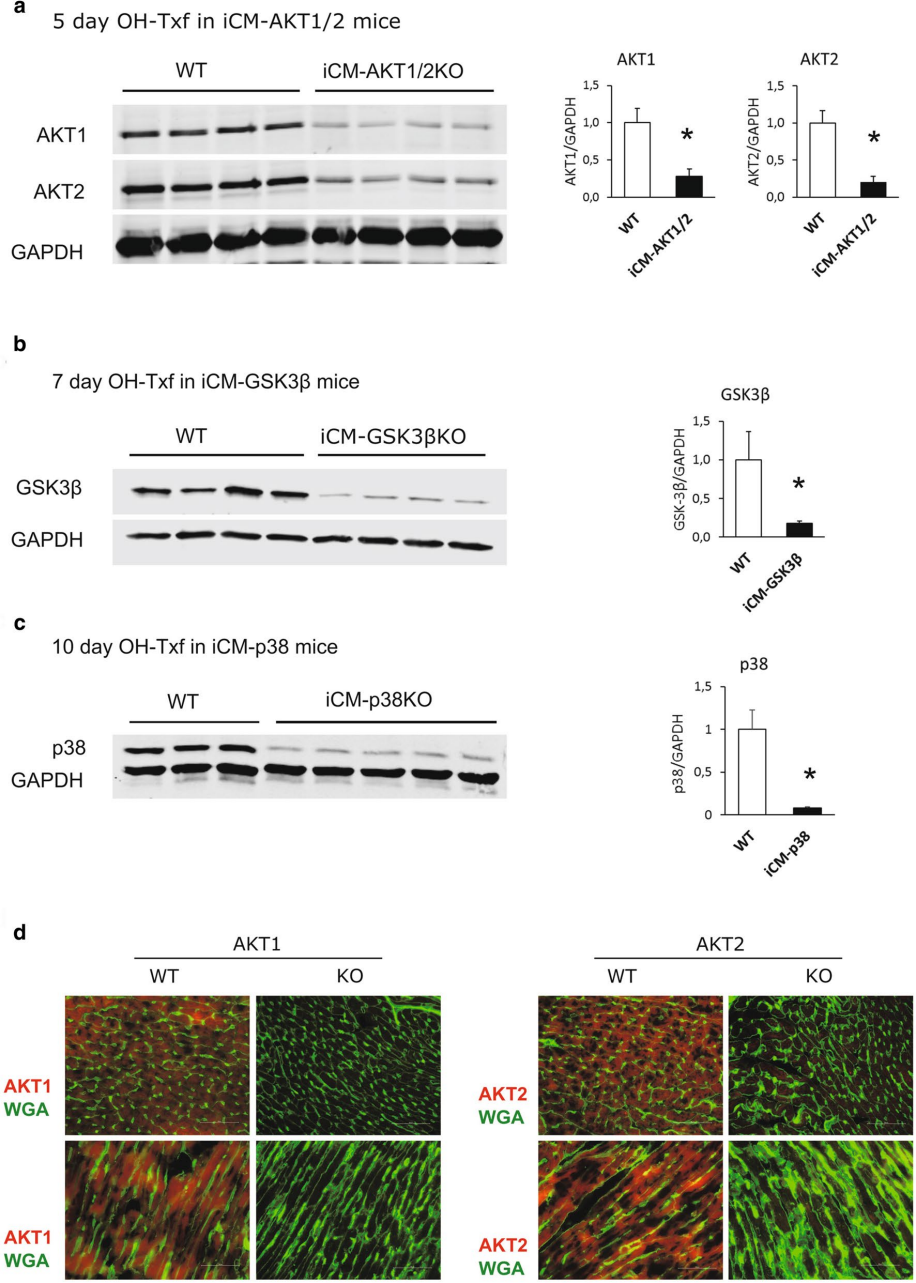
OH-Txf causes efficient gene deletion in floxed αMHC-MerCreMer mice. Representative western blots and summarized data of **a** Akt1 and Akt2, **b** GSK-3β, and **c** p38 of hearts from iCM-Akt1/2KO, iCM-GSK3βKO, and iCM-p38KO mice, respectively, are shown. Cardiomyocyte restricted gene deletion was initiated by intraperitoneal application of 20 mg/kg OH-Txf on 5, 7 or 10 consecutive days as indicated. **d** Representative immunofluorescence stainings of cross sectioned (upper panel) and longitudinal slices (lower panel) of hearts from iCM-Akt1/2 knock out mice (KO) or WT mice are shown. Administration of 20 mg/kg OH-Txf for five consecutive days resulted in loss of both Akt1 (left panel) and Akt2 (right panel) in cardiomyocytes. Data are normalized to WT values and presented as mean ± SD. *p < 0.05 vs WT (unpaired, two-tailed student’s *T*-test)

First, we aimed to demonstrate the efficiency of our OH-Txf treatment protocol to induce gene deletion in combination with the αMHC-MerCreMer/loxP system. Western blot quantification showed that administration of OH-Txf to iCM-Akt1/2 mice for five consecutive days resulted in a reduction of Akt1 and Akt2 protein expression by 70 and 80%, respectively (Fig. 1a). As the remaining Akt-isoform signals result from non-cardiomyocytes, these data further emphasize the effective protein depletion of this DNA editing method. In addition, administration of OH-Txf to iCM-Gsk3β mice for 7 days, or to iCM-p38 mice for 10 days initiated a strong reduction in protein expression of Gsk3β (Fig. 1b) or p38 (Fig. 1c), respectively. Taken together, OH-Txf in combination with the αMHC-MerCreMer/loxP system is an effective method for inducible, cardiomyocyte-restricted DNA editing in adult mice.

Next, we performed immunohistological stainings of tissue slices from iCM-Akt1/2WT mice to visualize the homogeneity of the protein depletion. Our results demonstrated the presence of both Akt1 and 2 in all cardiomyocytes of OH-Txf treated WT animals (Fig. 1d). In contrast, a homogeneous loss of both Akt1 as well as Akt2 signal was detected in cardiomyocytes from knockout animals after OH-Txf treatment for 5 days clearly demonstrating an efficient recombination after OH-Txf treatment in all cardiomyocytes.

As there is some evidence that a single injection of 40 mg/kg tamoxifen is efficient to induce loxP-mediated recombination [18, 24], we performed additional explorative experiments. Here, iCM-Akt1/2KO mice received a single i.p. injection of 40 mg/kg tamoxifen or OH-Txf. Subsequently, cardiac tissue homogenates were analysed by western blot for determination of protein depletion efficiency. Both tamoxifen and OH-Txf caused a reduction in Akt1 as well as Akt2 protein levels (supplementary material, *Fig. S1a*). However, the single injection of tamoxifen caused a reduction of both Akt1 und Akt2 of about 50% compared to untreated controls, and the single injection of OH-Txf resulted in a depletion efficiency of about 65%. These efficiency levels are clearly below the ones observed after the 5 day injection protocol with 20 mg/kg OH-Txf described above (indicated as dotted lines in *Fig. S1a*). Taken together, these explorative data indicate that (1) a robust protein depletion can be achieved using a single-injection protocol, (2) the depletion efficiency did not reach the level that was observed using the 5 day injection protocol with 20 mg/kg OH-Txf, and 3) OH-Txf might be more efficient in protein depletion than tamoxifen.

### OH-Txf treatment does neither disturb left ventricular function nor cardiac energy status in αMHC-MerCreMer mice

**Fig. 2:**
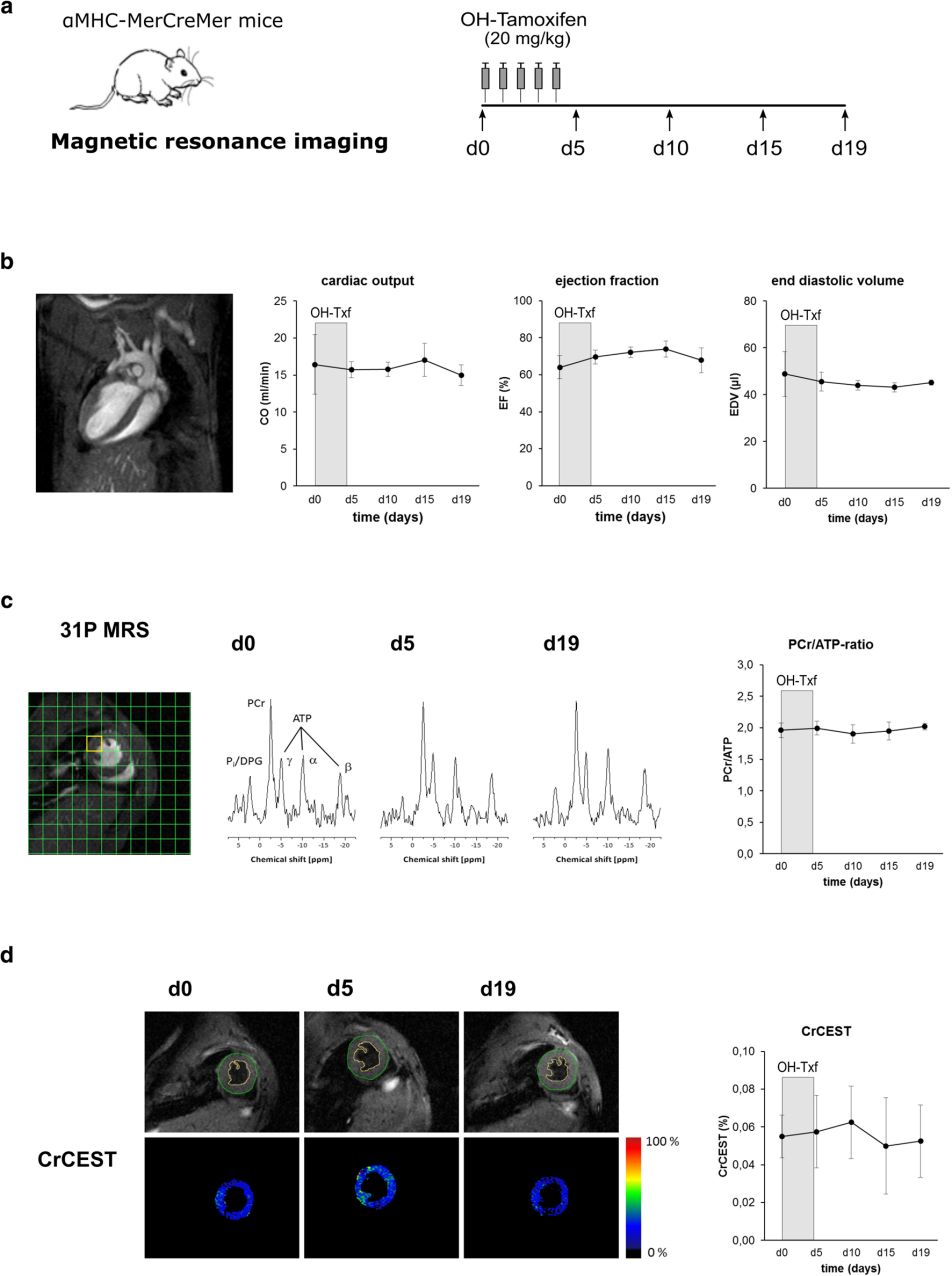
OH-Txf treatment does disturb neither cardiac function nor cardiac energetic status in αMHC-MerCreMer mice. **a** Experimental protocol; αMHC-MerCreMer mice received 20 mg/kg 4-hydroxytamoxifen (OH-Txf) intraperitoneally for 5 consecutive days. Data were obtained before (d0) and 5, 10, 15, and 19 days after the first OH-Txf injection, respectively. **b** Cardiac function was determined by MR imaging. An example image (left), and summarized data of cardiac output (CO), ejection fraction (EF), and end diastolic volume (EDV) are shown. **c** Cardiac energy status was determined using ^31^P MR spectroscopy. MR spectra were obtained from a voxel of the left ventricular free wall (yellow square in the example image; left). Example spectra from d0, d5, and d19 (middle), and summarized data of PCr/ATP ratios (right) are shown. **d** Creatine formation was assessed by CrCEST; example images from d0, d5, and d19 (left), and summarized data (right) are shown. Data are presented as mean ± SD. *n* = 4; *p < 0.05 vs d0 (one-way repeated-measure ANOVA, followed by Tukey’s post hoc test)

To systematically examine potential side effects of the αMHC-MerCreMer/loxP system during and after OH-Txf on cardiac function, we treated αMHC-MerCreMer mice with OH-Txf for 5 consecutive days and performed multi-parametric MRI for analysis of cardiac function and characterization of cardiac tissue (Fig. 2a).

During and after OH-Txf treatment, body weight and heart rates were comparable between the different days of analysis (supplementary material, *Tab. S1*). The MRI-based analysis of left ventricular function showed at d0, i.e. before the start of OH-Txf treatment, a CO of 16.4 ± 4.0 ml/min, an EF of 64.0 ± 6.2%, and an EDV of 48.8 ± 9.6 µl (Fig. 2b). The treatment with OH-Txf did not disturb CO during the observational period of 19 days after the start of OH-Txf treatment. Furthermore, we did not detect any deleterious effect of OH-Txf on EF or EDV indicating that OH-Txf treatment had no cardiac side effects, and does not cause a cardiomyopathic phenotype or cardiac dilatation (Fig. 2b).

Cre-mediated side effects of tamoxifen treatment have been described also to affect cardiac energy status [12]. Therefore, we performed ^31^P MR spectrometry-based analysis of cardiac PCr/ATP ratios to investigate cardiac energetics. The PCr/ATP ratio at d0, i.e. before OH-Txf treatment, was 1.96 ± 0.12 (Fig. 2c). PCr/ATP ratio was affected neither at d5, nor at d10, d15 or d19 compared to d0 values indicating that OH-Txf treatment of αMHC-MerCreMer mice has no side effect on cardiac energetic status. This finding is supported by the fact that also creatine formation assessed by CrCEST is not disturbed during the observational period of 19 days (Fig. 2d).

Interestingly, the absence of functional or energetic disturbances was not only detected in αMHC-MerCreMer mice, but also in another experimental series, in that OH-Txf was administered for 5 consecutive days to Cre-negative mice further supporting that OH-Txf does not cause unwanted cardiac side effects (supplementary material, *Fig. S2*).

As described above, a single injection of 40 mg/kg tamoxifen has been described to effectively initiate DNA recombination, we aimed to test whether this injection protocol as well as the injection of 40 mg/kg OH-Txf result in disturbances in cardiac function. For this, tamoxifen or OH-Txf was injected into αMHC-MerCreMer mice, and cardiac function was determined by echocardiography at d0, d3 and d5, respectively. The injection of 40 mg/kg OH-Txf did not impair cardiac function (supplementary material, Fig. S1b). In contrast, cardiac function was clearly impaired after a single injection of tamoxifen, especially seen in an increase in EDV indicating ventricular dilatation.

### OH-Txf treatment does not induce tissue fibrosis or causes cardiac lipid accumulation and/or tissue oedema

**Fig. 3:**
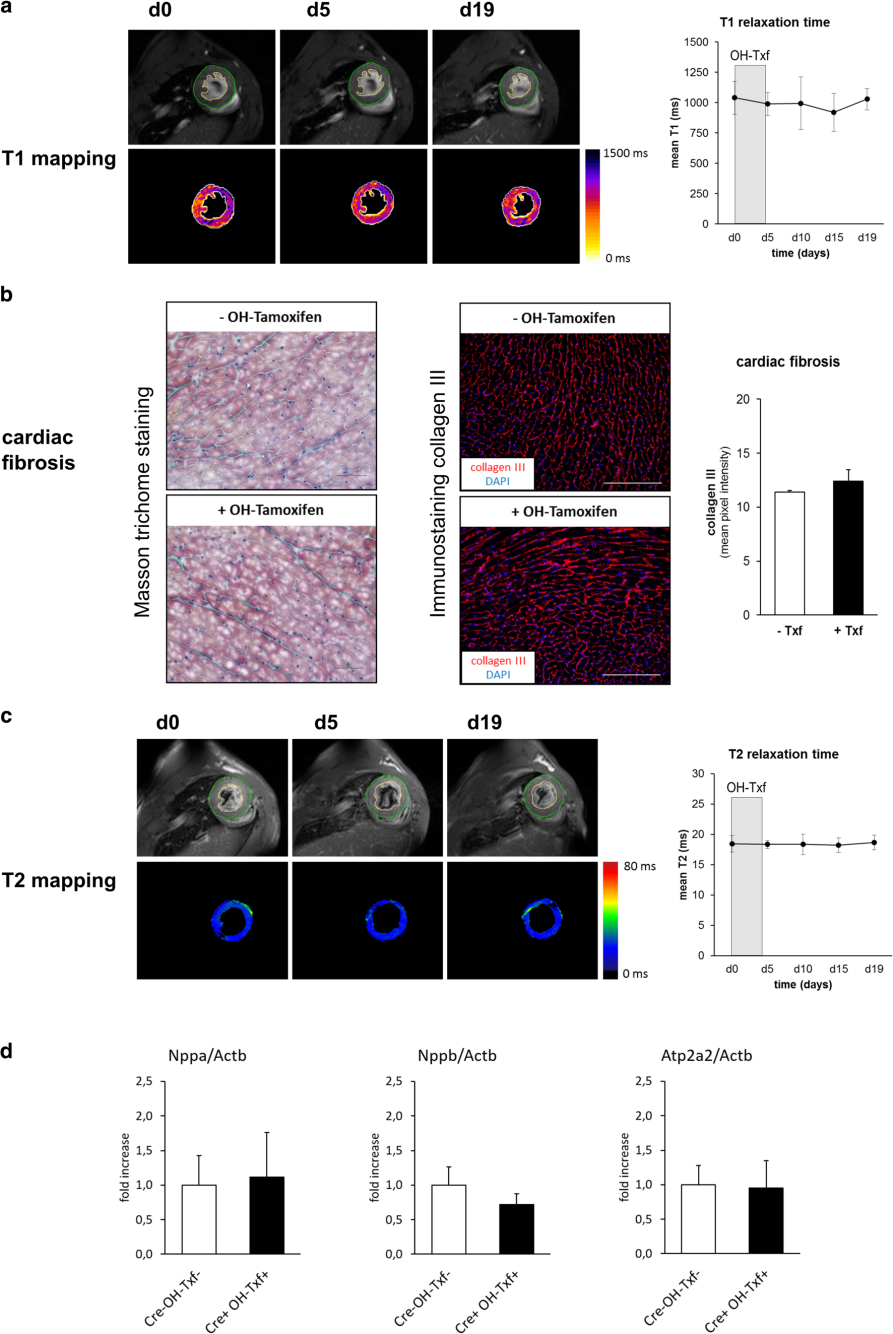
Tissue characterization of OH-Txf treatment does not induce tissue fibrosis or cause lipid accumulation and/or tissue oedema **a** Example T1 maps of hearts from OH-Txf treated αMHC-MerCreMer mice at d0, d5, and d19 (left), and summarized data of mean T1 relaxation time (right) are shown. **b** Cardiac fibrosis was assessed by Masson trichrome staining (left) or immunohistological collagen III staining (right) at d19 of untreated (-OH-Tamoxifen) or OH-Txf treated (+ OH-Tamoxifen) hearts of αMHC-MerCreMer mice. Example images for Masson trichrome staining and collagen III staining as well as summarized data of collagen III content (right) are shown. Scale bare represents 50 µm (Masson trichrome) or 200 µm (collagen III). **c** Example T2 maps of hearts from OH-Txf treated αMHC-MerCreMer mice at d0, d5, and d19 (left), and summarized data of mean T2 relaxation time (right) are shown (*n* = 4). **d** Relative mRNA expression of *Nppa*, *Nppb* and *Atp2a2* were determined as marker genes for cardiomyopathy by qPCR in hearts of αMHC-MerCreMer mice at d19 after OH-Txf treatment for 5 days (Cre + OH-Txf + ; *n* = 5), and Cre-negative mice that did not receive OH-Txf (Cre- OH-Txf-; *n* = 6). The results were normalized to *Actb* mRNA expression, and x-fold induction was calculated relative to the expression in the Cre- OH-Txf- group. Data are presented as mean ± SD. *p < 0.05 vs d0 (one-way repeated-measure ANOVA, followed by Tukey’s post hoc test) or unpaired, two-tailed student’s *T*-test

As the development of cardiac fibrosis is accompanied by alterations in T1 relaxation time [13], MR relaxometry can be used as non-invasive indicator of cardiac fibrosis. Here, we analysed T1 relaxation time in αMHC-MerCreMer mice before and after OH-Txf treatment. At d0, mean T1 time was 1040 ± 135 ms, and no effect of OH-Txf treatment on mean T1 time was observed during the observational period of 19 days suggesting that no cardiac fibrosis developed as consequence of the OH-Txf treatment (Fig. 3a). This finding was confirmed by Masson trichrome staining as well as by immunohistological stainings for collagen III of cardiac tissue slices showing no difference between OH-Txf treated and non-treated hearts (Fig. 3b). We further performed cardiac tissue characterization by analysing T2 relaxation time as sensitive readout for pathological tissue alterations including oedema or lipid accumulation [17, 27]. Here, mean T2 relaxation time was not altered during or after OH-Txf treatment (Fig. 3c). In addition, OH-Txf treatment of αMHC-MerCreMer mice for 5 days did not affect expression of *Nppa*, *Nppb* as well as *Atp2a2* compared to untreated Cre-negative mice further supporting the absence of a cardiomyopathic phenotype (Fig. 3d). Taken together, these results from the multi-parametric MR imaging-based tissue characterization in combination with the histological results did not show any indication of Cre-mediated cardiac side effects after OH-Txf treatment.

### Long-term effects of OH-Txf treatment on cardiac function in αMHC-MerCreMer mice

**Fig. 4:**
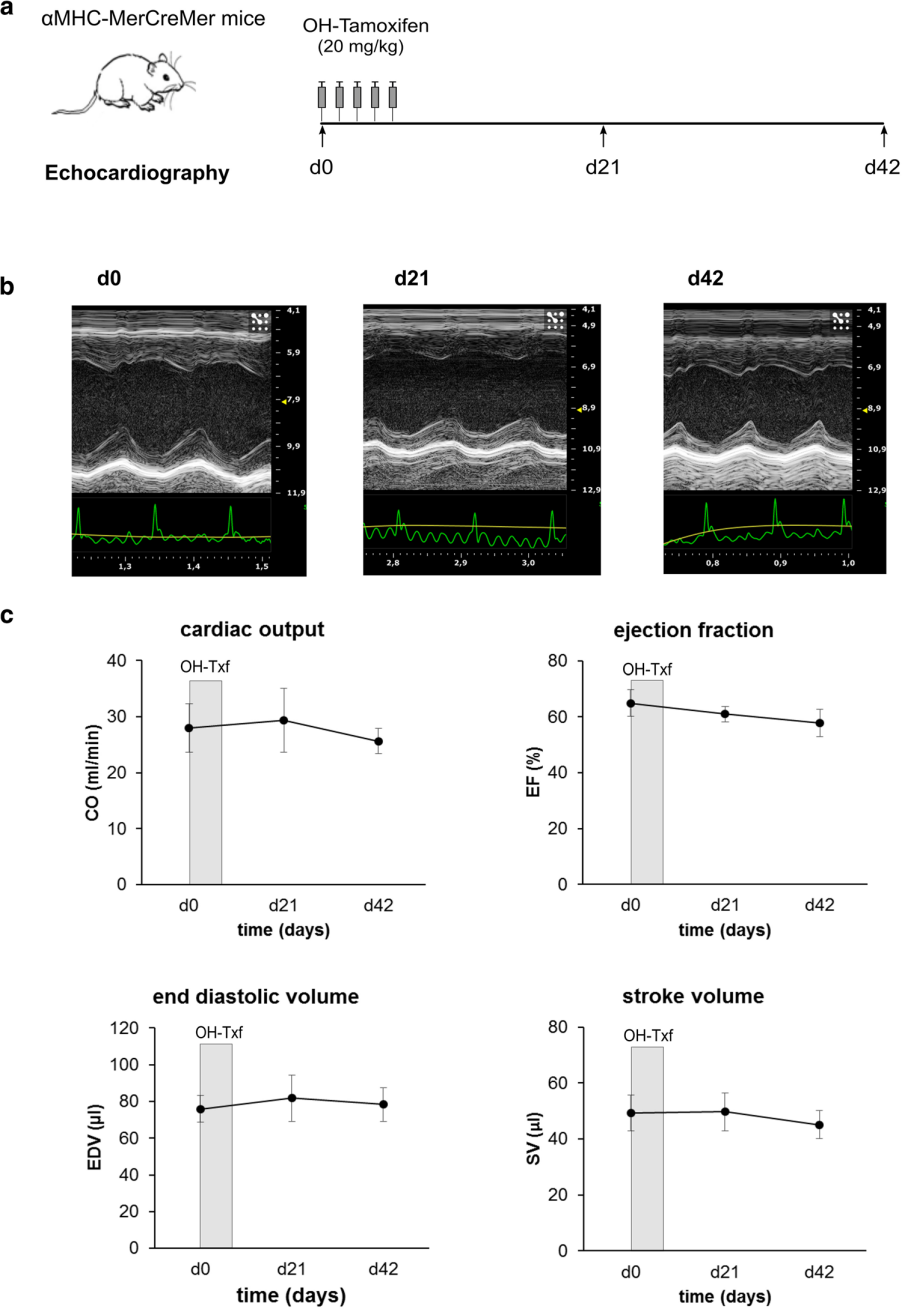
Long-term effects of OH-Txf treatment on cardiac function in αMHC-MerCreMer mice. **a** Experimental protocol; αMHC-MerCreMer mice received 20 mg/kg 4-hydroxytamoxifen (OH-Txf) intraperitoneally for 5 consecutive days. Cardiac function was analysed by echocardiography before (d0) and 21 and 42 days after the first OH-Txf injection, respectively. **b** Representative parasternal short-axis M-mode views of d0 (left), d21 (mid), and d42 (right) recordings. Scaling of the x-axis in seconds, scaling of the y-axis: mm. **c** Summarized data for cardiac output (CO), ejection fraction (EF), end diastolic volume (EDV), and stroke volume (SV). Data are presented as mean ± SD. *n* = 5; *p < 0.05 vs d0 (one-way repeated-measure ANOVA, followed by Tukey’s post hoc test)

To examine whether OH-Txf-treated αMHC-MerCreMer mice develop a cardiomyopathic phenotype at later time points than the investigated 19 day period of the MR investigations above, we treated an additional group of mice with OH-Txf for 5 consecutive days and analysed cardiac function by echocardiography for up to 6 weeks (Fig. 4a). During and after OH-Txf treatment, body weight and heart rates were comparable between the different days of analysis (supplementary material, *Tab. S2*). The mice showed at d0, i.e. before the start of OH-Txf treatment, a CO of 28.0 ± 4.3 ml/min, an EF of 64.9 ± 4.7%, an EDV of 75.8 ± 7.2 µl, and a SV of 49.3 ± 6.3 µl (Fig. 4b, c). The application of OH-Txf did not affect CO, EF, EDV or SV, neither at d21 nor at d42 (Fig. 4c). Additionally, no effects on wall thickness and dimensions of the left ventricle, on heart weight or on heart-to-body weight ratios were detected (Supplementary material online, Fig. S3). These data indicate that administration of OH-Txf for 5 consecutive days does not initiate a transient or permanent cardiomypathic phenotype in αMHC-MerCreMer mice.

### 10 days OH-Txf treatment and cardiac function in αMHC-MerCreMer mice

**Fig. 5:**
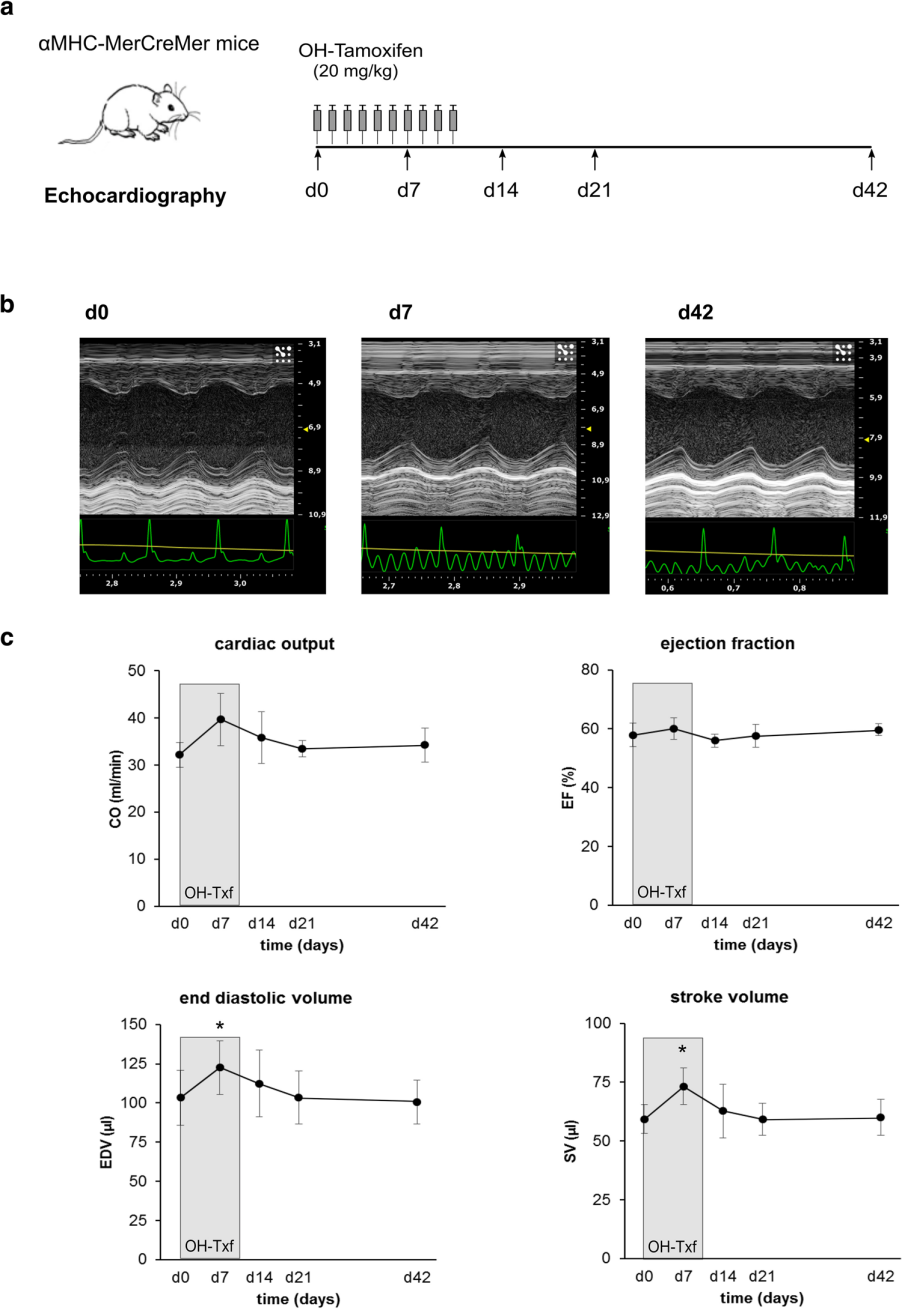
4-Hydroxytamoxifen treatment for 10 days does not induce cardiomyopathy. **a** Experimental protocol; αMHC-MerCreMer mice received 20 mg/kg 4-hydroxytamoxifen (OH-Txf) intraperitoneally for 10 consecutive days. Cardiac function was analysed by echocardiography before (d0) and 7, 14, 21 and 42 days after the first OH-Txf injection, respectively. **b** Representative parasternal short-axis M-mode views of d0 (left), d7 (mid), and d42 (right) recordings. Scaling of the x-axis in seconds, scaling of the y-axis: mm. **c** Summarized data for cardiac output (CO), ejection fraction (EF), end diastolic volume (EDV), and stroke volume (SV). Data are presented as mean ± SD. *n* = 4; *p < 0.05 vs d0 (one-way repeated-measure ANOVA, followed by Tukey’s post hoc test)

Finally, we sought to determine whether a prolonged administration protocol of OH-Txf is accompanied by defects in cardiac function. For this, we treated αMHC-MerCreMer mice for 10 consecutive days with OH-Txf (Fig. 5a). During and after OH-Txf treatment, body weight and heart rates were comparable between the different days of analysis (supplementary material, Table S2). Before the start (d0) of treatment, the CO was 32.2 ± 2.7 ml/min, the EF 58.0 ± 4.0%, the EDV 103.4 ± 17.4 µl, and the SV 59.3 ± 6.1 µl (*Fig. **5**b-c*). The application of OH-Txf caused at d7 a transient increase in EDV (122.7 ± 17.1 µl). However, this increase cannot be interpreted as a sign of reduced cardiac function as it was accompanied by an increased SV (73.3 ± 7.8 µl). In line with this conclusion, both CO and EF were stable at d7 compared to d0. At all other time points, no effects of OH-Txf treatment on functional parameters were observed (Fig. 5c). In addition, 10-day OH-Txf treatment had no effect on wall thickness and dimensions of the left ventricle, on heart weight or on heart-to-body weight ratios (Supplementary material online, *Fig. S4*). Taken together, also a prolonged OH-Txf treatment did not induce a cardiomyopathic phenotype of αMHC-MerCreMer mice.

## Discussion

The αMHC-MerCreMer/loxP system is routinely used for cardiac restricted, temporally controlled DNA editing in cardiac tissue, e.g. for protein depletion, as multiple mouse lines with genes of interest that are flanked by loxP sites are available. On the other hand, the applicability of this approach has been questioned because Cre-mediated cardiac off-target effects have been reported in some studies, in which the Cre-recombinase was activated by the application of tamoxifen [3, 20, 24].

Tamoxifen was administered in previous studies showing adverse effects on cardiac function at doses of 40 mg/kg per day or higher. For example, Koitabashi et al. reported that a dose of 80 mg/kg tamoxifen per day was required for an efficient gene knockdown, but this was associated with a dramatic decrease in EF suggesting a cardiomyopathy with left ventricular dilatation and a 60% mortality due to severe cardiomyopathy in αMHC-MerCreMer mice [20]. Hall et al. reported next to deterioration of cardiac contractile function also an increase in ventricular wall thickness after administration of 40 mg/kg tamoxifen in αMHC-MerCreMer mice [12]. Next to the tamoxifen-induced adverse effects on cardiac function, also side effects of tamoxifen on the bioenergetic status of cardiomyocytes, e.g. a reduced mitochondrial ATP production, have been described [2, 12].

Interestingly, OH-Txf, the active metabolite of tamoxifen, is often used for Cre-activation in cell culture models, but little is known about the required treatment dosage and duration for an efficient αMHC-MerCreMer/loxP-based DNA editing in vivo.

The present study and prior investigations of our group demonstrate that the use of OH-Txf induces highly efficient DNA editing at an OH-Txf dose of 20 mg/kg per day [15, 26]. Importantly, this dose of OH-Txf did not cause any mortality in αMHC-MerCreMer mice, and we did not detect adverse effects of this OH-Txf treatment protocol related to cardiac function, hypertrophy, dilation, expression of heart failure markers, metabolism, and fibrosis.

In the present study, the intraperitoneal injection of OH-Txf for 5–10 consecutive days yields a high degree of gene depletion efficiency in three different floxed mouse lines, all of them demonstrating no deterioration of cardiac function measured by echocardiography. The only exception was a transient increase in EDV on day 7 of the 10-day treatment protocol which might potentially indicate a restricted function. However, we interpret that this increase in EDV is rather caused by experimental variation than by a real cardiac dysfunction as at the same time, both cardiac output and stroke volume were increased, and ejection fraction was unchanged.

As a cardiomyopathy is often associated with decreased cardiac energy status and Txf administration affected cardiac energetics [2, 12], we investigated also the effects of OH-Txf treatment on cardiac bioenergetics using in vivo ^31^P MR spectroscopy and CrCEST [7, 29]. Our results clearly show that OH-Txf has no adverse effects on cardiac energy status as both cardiac PCr/ATP ratios and creatine levels were stable during and after the OH-Txf treatment.

MR imaging was also used for additional tissue characterization. MR relaxometry [5, 11] revealed no indication for the development of cardiac fibrosis after OH-Txf treatment as seen by unchanged T1 relaxation time, which was in line with histological analysis. Furthermore, the mean T2 time in our experiments was unchanged, indicating that the development of tissue oedema or cardiac lipid accumulation due OH-Txf treatment is unlikely. The latter finding is particularly important, because OH-Txf is dissolved in peanut oil, and therefore, a high amount of lipids is administered, which might lead to cardiac lipid accumulation, which, in turn, could promote cardiac dysfunction. Taken together, OH-Txf application is a safe approach to induce gene deletion, even if a locus-specific prolonged treatment is required to efficiently excise floxed DNA segments in case of, e.g. poor accessibility of the loxP sites, due to chromatin structure.

In view of the side effects of prolonged Txf treatment reported in some studies, several alternative strategies have been developed to overcome Txf-associated problems. Raloxifene is an alternative ER modulator which was initially shown to mediate gene deletion without inducing transient cardiomyopathy [20], but as reported later by the same group resulted in an insufficiently consistent gene deletion in the same floxed mouse line as in their initial study [21].

Oral application of tamoxifen citrate is discussed to overcome the problems of cardiomyopathy [19, 24], but also induced transient LV dilation in other studies [20]. Moreover, a single tamoxifen application has been shown to fully induce loxP-mediated recombination in αMHC-MerCreMer transgenic mice at the Serca2flox/flox mice [18] and a floxed tdTomato/Ai14 reporter gene [24]. This favourable approach failed in other studies [4], and tamoxifen is generally administered in for 4–5 days [1, 22], and even longer treatment protocols have been described [10, 23]. In addition, our explorative experiments suggested that the protein depletion efficiency after a single-injection protocol induces protein depletion less efficiently than the protocol with five repeated injections. However, we think that the efficiency of the protein depletion should to be tested for each gene locus or rather mouse strain to find the optimal injection protocol.

As further alternative strategy an approach for the safe induction of Cre-mediated recombination in the heart was developed based on adeno-associated viral (AAV) vectors achieving cardiomyocyte specificity by a combination of a preferred cardiac tropism of the selected AAV serotype in combination with a cardiomyocyte-specific promotor, such as the TnI promoter [9, 28, 32]. The advantage of the AAV vector-guided Cre expression is that it requires neither time-consuming breeding strategies nor Txf injection to delete a gene in the adult mouse heart. On the other hand, 5–10% of cells in the liver showed still a recombination [32], which demonstrates a lower specificity of the AAV-based recombination than the αMHCMerCreMer system. At the end, the choice of the methodological approach should be based on specific requirements of the planned experiments.

Taken together, there exists no unique “one-for-all” protocol for tamoxifen-inducible Cre-mediated gene deletion. However, the detailed functional, structural, and metabolic analysis presented in this work reports a comprehensive data set, which demonstrates that the use of OH-Txf for MerCreMer-activation avoids cardiac dysfunction caused by inducible, cardiomyocyte-restricted DNA recombination. This conclusion refers to standard five-day OH-Txf treatment as well as to prolonged ten-day protocols.

## Supplementary Information

Below is the link to the electronic supplementary material.

Supplementary file1 (DOCX 2304 KB)
