# Supplementary material for: 4-hydroxytamoxifen does not deteriorate cardiac function in cardiomyocyte-specific MerCreMer transgenic mice
Source: Basic Res Cardiol. 2021 Feb 5;116(1):8. doi: 10.1007/s00395-020-00841-9 (PMC7864833; doi:10.1007/s00395-020-00841-9)
Supplement: Supplementary file 1 — Supplementary file1 (DOCX 2304 KB) [file 395_2020_841_MOESM1_ESM.docx]

**Online Supplementary Material**

4-hydroxytamoxifen does not deteriorate cardiac function in cardiomyocyte-specific MerCreMer transgenic mice

Andre Heinen^1^, Stefanie Gödecke^1^, Ulrich Flögel^2^, Dominika Miklos^1^, Katharina Bottermann^1^, Andre Spychala^1^, Axel Gödecke^1^

^1^ Institut für Herz- und Kreislaufphysiologie, Universitätsklinikum, Heinrich-Heine-Universität Düsseldorf, 40225 Düsseldorf, Germany

^2^ Institut für Molekulare Kardiologie, Universitätsklinikum, Heinrich-Heine-Universität Düsseldorf, 40225 Düsseldorf, Germany

Corresponding author

Axel Gödecke, Ph.D.

Institut für Herz- und Kreislaufphysiologie

Universitätsklinikum, Heinrich-Heine-Universität Düsseldorf

Universitätsstraße 1, 40225 Düsseldorf, Germany

Fax: +49 (0)211 81 016 12675

Tel.: +49 (0)211 81 11612670

E-Mail: axel.goedecke@uni-duesseldorf.de


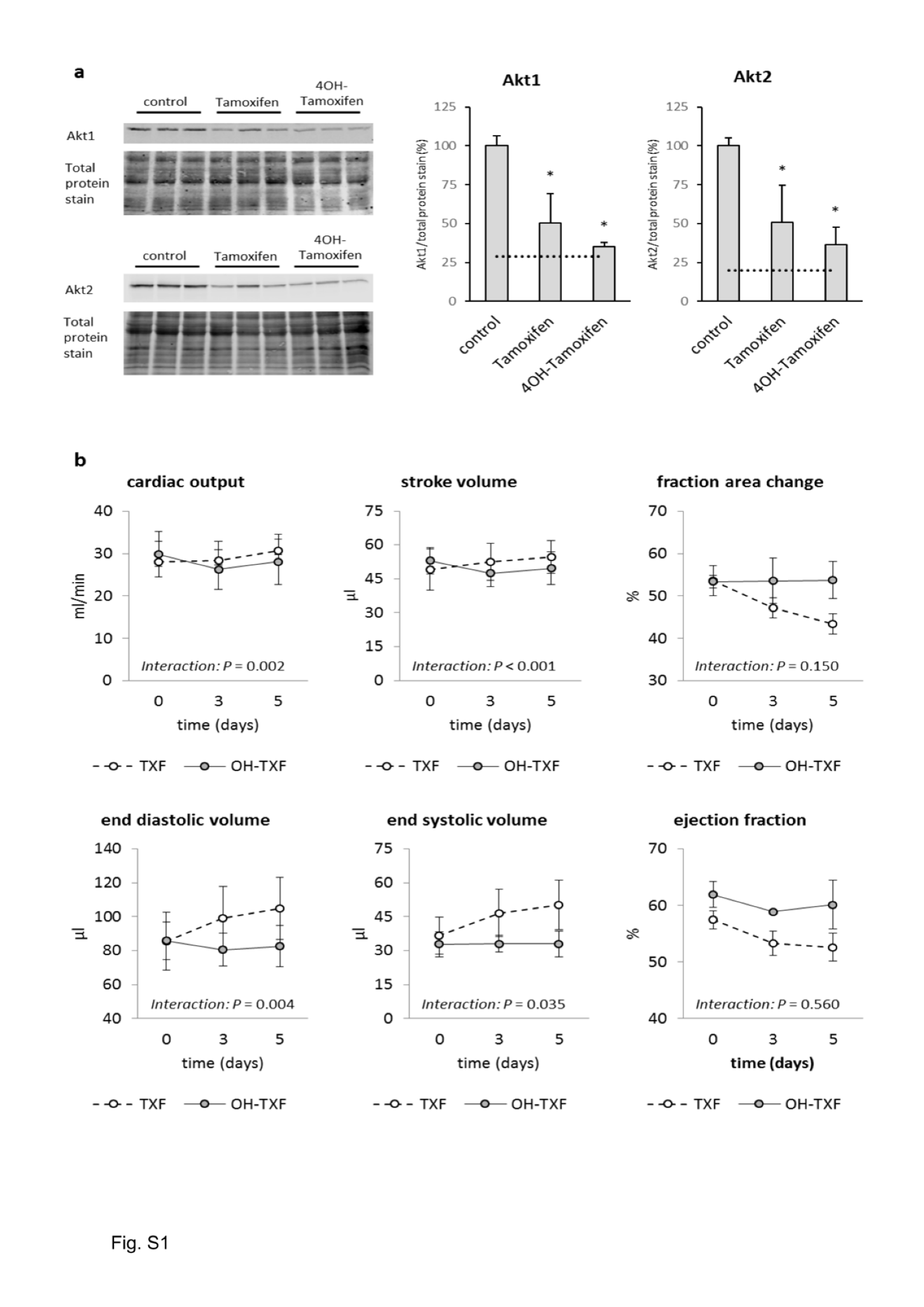


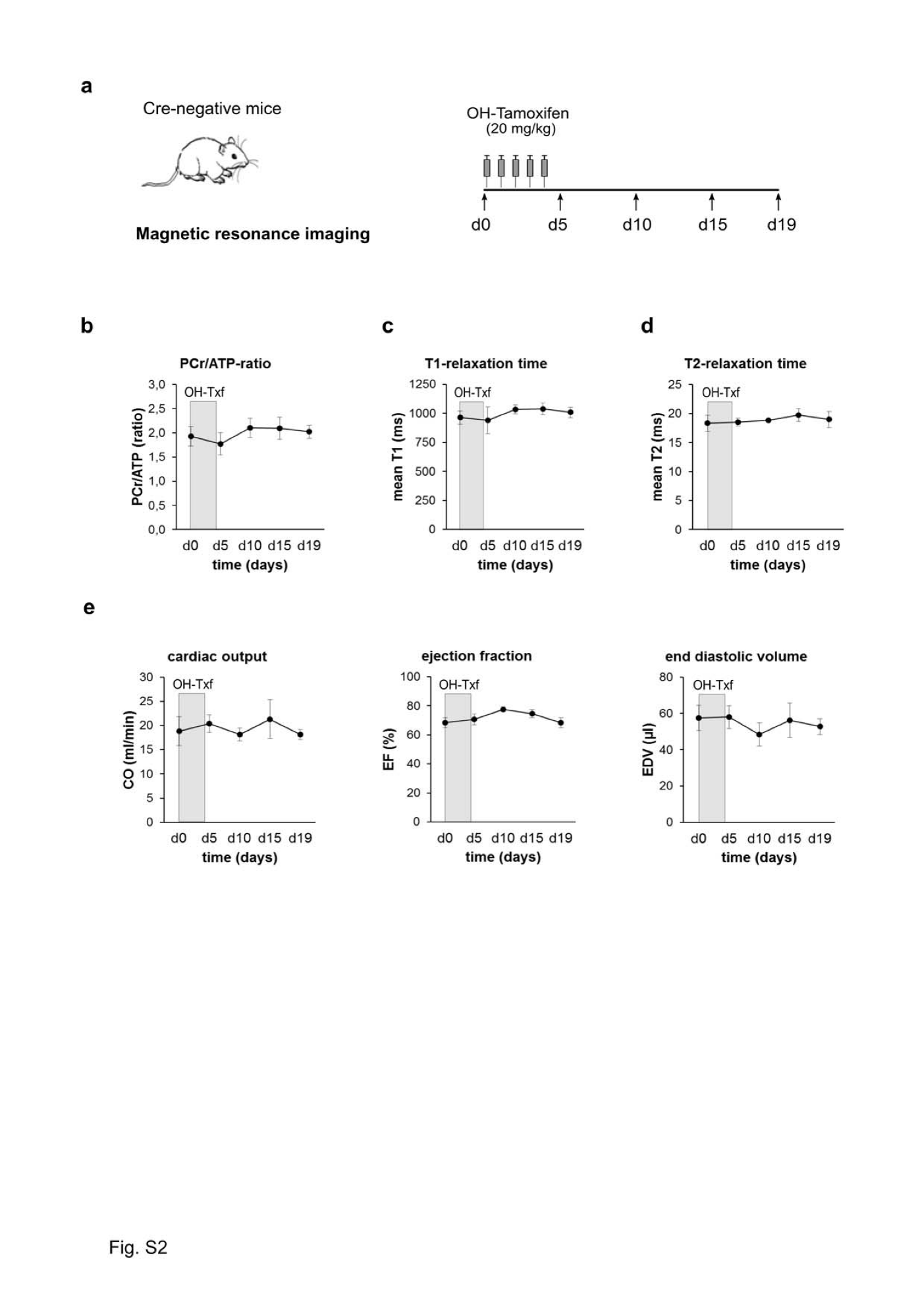


**
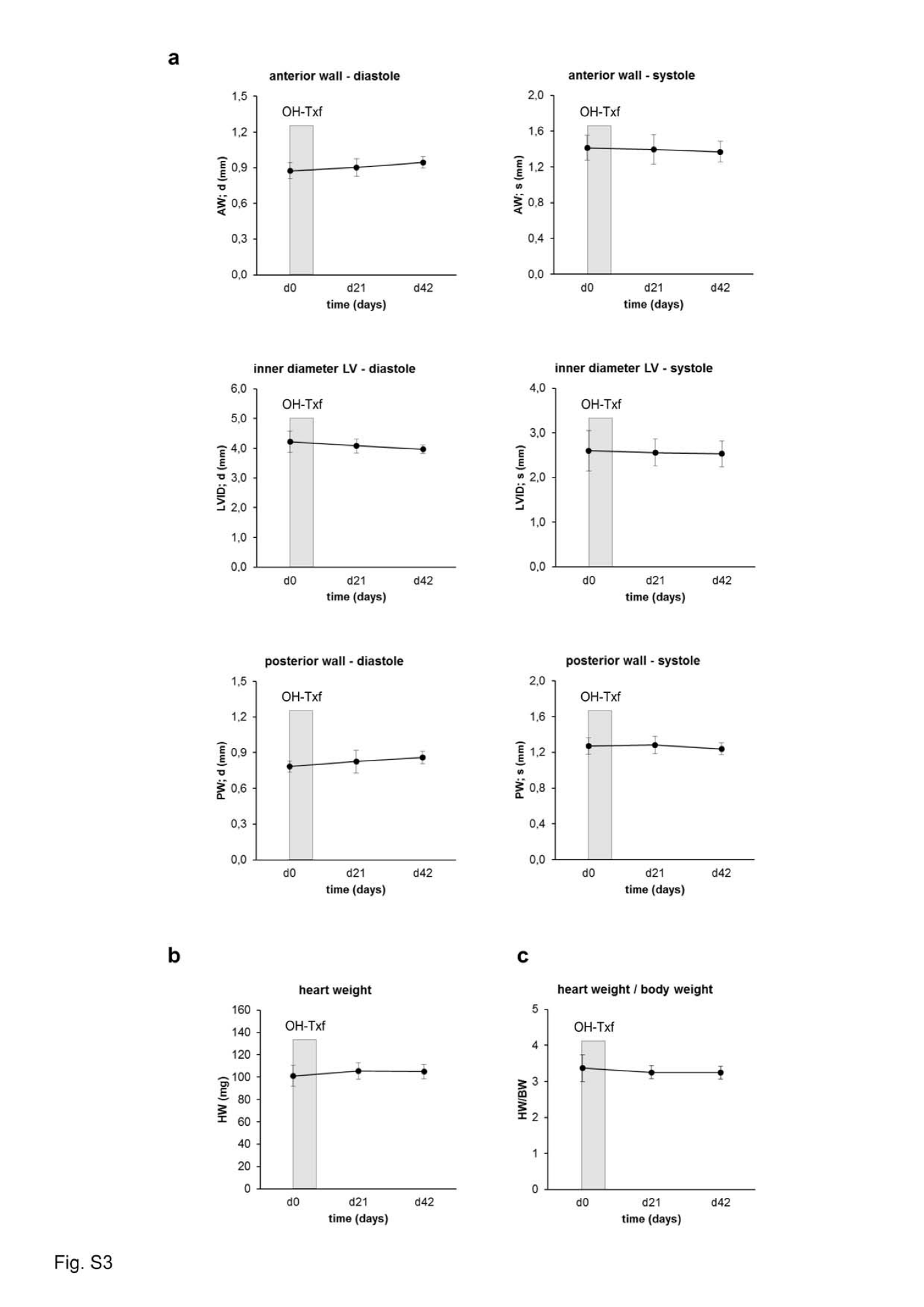
**

**
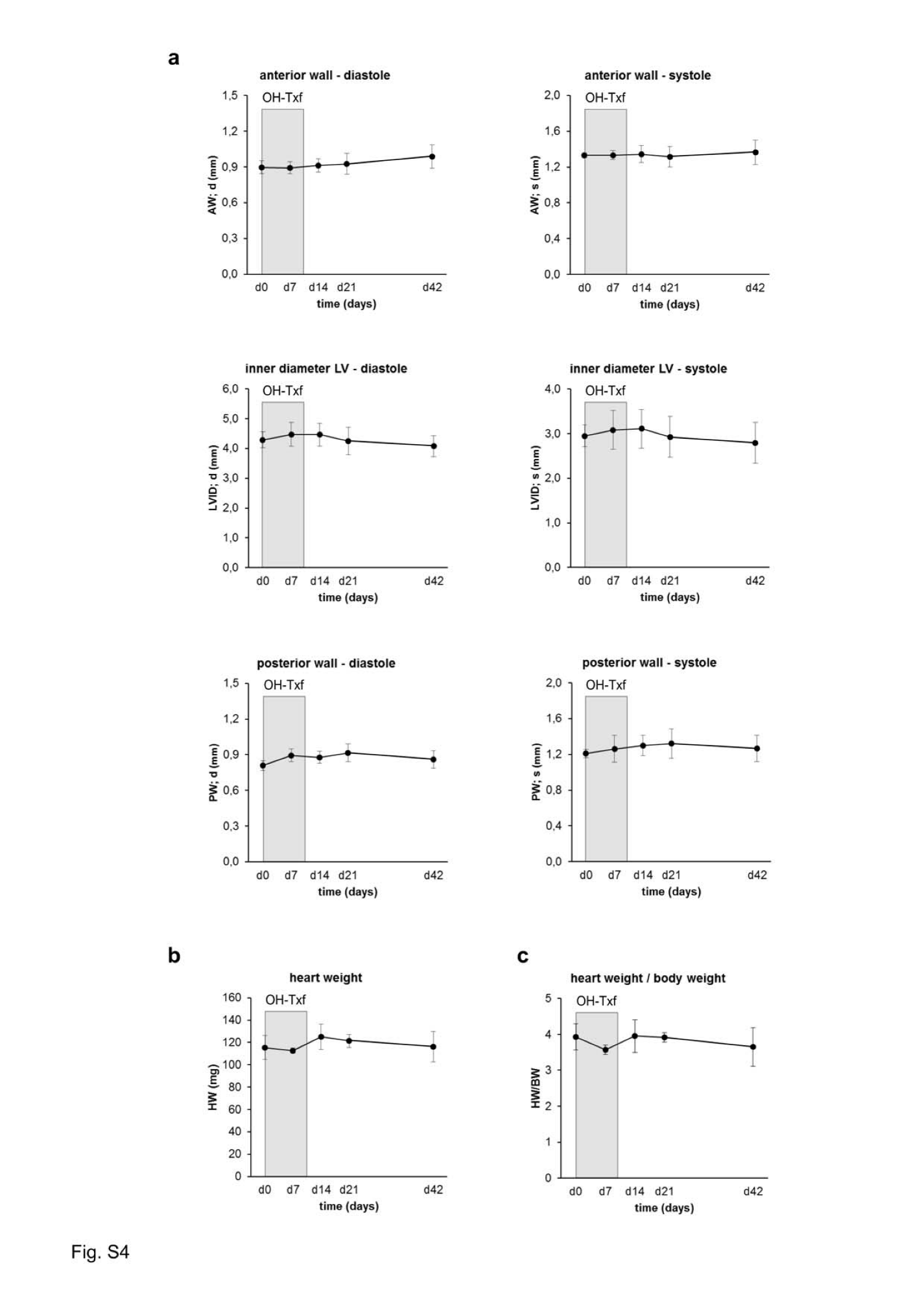
**

**Figure legends**

**Fig. S1** Protein depletion efficiency and cardiac side-effects of single-injection protocols

(a) Representative western blots and summarized data of Akt1 and Akt2 of hearts from iCM-Akt1/2KO are shown. Cardiomyocyte restricted gene deletion was initiated by a single intraperitoneal injection of 40 mg/kg tamoxifen or OH-Txf. Controls did not receive an injection. The dashed lines indicate the level of protein depletion that was detected after administration of 20 mg/kg OH-Txf for five consecutive as shown in figure 1. Data are normalized to control values and presented as mean ± SD. * p<0.05 vs control (n = 3 per group; one-way ANOVA followed by Tukey’s post hoc test).

(b) Summarized data for parameter of cardiac function. αMHC-MerCreMer mice received either 40 mg/kg tamoxifen (TXF) or OH-Txf as single intraperitoneal injection. Cardiac function was analysed by echocardiography before (0) and 3 and 5 days after the injection, respectively. Data are presented as mean ± SD. n = 3; two-way repeated measure ANOVA.

**Fig. S2** Cre-independent effects of 4-hydroxytamoxifen treatment on cardiac energetics, tissue characterization, and function

(a) Experimental protocol; Cre-negative C57Bl/6J mice received 20 mg/kg 4-hydroxytamoxifen (OH-Txf) intraperitoneally for 5 consecutive days. Summarized data (b) of cardiac energetics assessed by ^31^P magnetic resonance spectroscopy, and (c-d) MR relaxometry based T1-, and T2-relaxation time. (e) Summarized data for cardiac output (CO), ejection fraction (EF), and end diastolic volume (EDV). Data were obtained before (d0) and 5, 10, 15, and 19 days after the first OH-Txf injection, respectively. Data are presented as mean ± SD. n = 3; one-way repeated measure ANOVA, followed by Tukey’s post hoc test.

**Fig. S3** Absence of adverse effects of OH-Txf treatment for 5 days on left ventricular dimensions and heart weight

αMHC-MerCreMer mice received 20 mg/kg 4-hydroxytamoxifen (OH-Txf) intraperitoneally for 5 consecutive days. Cardiac function was analysed before OH-Txf injection (d0), as well as 21 and 42 days after the first injection, respectively. Summarized echocardiographic data for (a) anterior wall thickness (AW), left ventricular inner diameter (LVID), and posterior wall thickness (PW) during diastole (left) and systole (right), for (b) heart weight, and (c) heart weight to body weight ratio. Data are presented as mean ± SD. n = 5; one-way repeated measure ANOVA, followed by Tukey’s post hoc test.

**Fig. S4** Absence of adverse effects of OH-Txf treatment for 10 days on left ventricular dimensions and heart weight

αMHC-MerCreMer mice received 20 mg/kg 4-hydroxytamoxifen (OH-Txf) intraperitoneally for 5 consecutive days. Cardiac function was analysed before OH-Txf injection (d0), as well as 7, 14, 21 and 42 days after the first injection, respectively. Summarized echocardiographic data for (a) anterior wall thickness (AW), left ventricular inner diameter (LVID), and posterior wall thickness (PW) during diastole (left) and systole (right), for (b) heart weight, and (c) heart weight to body weight ratio. Data are presented as mean ± SD. n = 4; one-way repeated measure ANOVA, followed by Tukey’s post hoc test.

**Supplemental tables**

| **Table S1:** MRI series | | | | | | | | | | | |  | | | |
| --- | --- | --- | --- | --- | --- | --- | --- | --- | --- | --- | --- | --- | --- | --- | --- |
| **5 day OH-Txf series MRI** | | | |  | |  |  | | |  | | | |  | |
|  | d0 | | d5 | | | | | d10 | | | d15 | | | | d19 |
| BW (g) | 27.9±1.6 | | 29.2±0.9 | | | | | 29.2±1.3 | | | 29.5±1.4 | | | | 29.3±1.1 |
| HR (bpm) | 523±22 | | 497±26 | | | | | 497±21 | | | 536±66 | | | | 489±41 |
| **Table S2:** Echocardiography series | | | | | | | | | | | | |  |  |  |
| **5 day OH-Txf series echocardiography** | | |  | |  | | | |  | | | |  |  |  |
|  | | d0 | | | d21 | | | | d42 | | | |  |  |  |
| BW (g) | | 30.1±1.7 | | | 32.5±3.3 | | | | 32.5±2.6 | | | |  |  |  |
| HR (bpm) | | 569±48 | | | 539±53 | | | | 570±22 | | | |  |  |  |
|  | |  | | |  | | | |  | | | |  |  |  |
| **10 day OH-Txf series echocardiography** | | |  | |  | | | |  | | | |  |  |  |
|  | | d0 | | | d21 | | | | d42 | | | |  |  |  |
| BW (g) | | 29.4±1.1 | | | 31.0±1.7 | | | | 31.9±1.6 | | | |  |  |  |
| HR (bpm) | | 545±23 | | | 569±34 | | | | 574±25 | | | |  |  |  |

BW, body weight; HW, Heart weight; HR, heart rate
